# The effectiveness of cinacalcet: a randomized, open label study in chronic hemodialysis patients with severe secondary hyperparathyroidism

**DOI:** 10.1080/0886022X.2018.1562356

**Published:** 2019-04-24

**Authors:** Paweena Susantitaphong, Somratai Vadcharavivad, Teerada Susomboon, Wanchana Singhan, Netsiri Dumrongpisutikul, Ketsuda Jakchairoongruang, Somchai Eiam-Ong, Kearkiat Praditpornsilpa

**Affiliations:** a Division of Nephrology, Department of Medicine, Faculty of Medicine, King Chulalongkorn Memorial Hospital, Chulalongkorn University, Bangkok, Thailand;; b Department of Pharmacy Practice, Chulalongkorn University, Bangkok, Thailand;; c Department of Radiology, Faculty of Medicine, Chulalongkorn University, Bangkok, Thailand

**Keywords:** Cinacalcet, chronic hemodialysis, severe secondary hyperparathyroidism

## Abstract

**Background:** Secondary hyperparathyroidism (SHPT) is associated with high incidences of cardiovascular disease, bone fracture, and mortality. This study was conducted to demonstrate the effectiveness of cinacalcet treatment on chronic kidney disease-mineral bone disorder (CKD-MBD) markers in chronic hemodialysis patients with severe SHPT.

**Methods:** In phase 1, 30 adult HD patients were randomized to cinacalcet or control groups for 12 weeks to explore the achievement of >30% reduction of iPTH. In phase 2, 45 patients were participated to further explore the effect of cinacalcet on CKD-MBD parameters for 24-week follow up and 12 additional weeks after cinacalcet discontinuation.

**Results:** In phase 1, the baseline serum iPTH levels were not different [1374 (955, 1639) pg/mL in the control group vs. 1191 (1005, 1884) pg/mL in the cinacalcet group], the percentage of patients achieving iPTH target were significantly higher in the treatment group [80% vs. 13%, *p* = .001]. In phase 2, the significant reductions of iPTH, FGF-23, tartrate-resistant acid phosphatase 5b, and slightly decreased size of parathyroid gland and stabilized vascular calcification were observed at 24-week follow up and markedly rebounded after discontinuation of cinacalcet.

**Conclusions:** The effectiveness of cinacalcet were still obviously demonstrated even in chronic HD patients with severe SHPT. In addition, the improvements of bone markers and FGF-23, and stabilization of vascular calcification were observed. Therefore, cinacalcet can provide salutary effects on CKD-MBD in severe SHPT and might be an initially effective PTH-lowering therapy prior to surgical parathyroidectomy as well as an alternative treatment in the patients unsuitable for surgery.

**Clinical trial registration:** ClinicalTrials.gov: NCT02056730. Date of registration: February 4, 2014.

## Introduction

Chronic kidney disease (CKD)-mineral bone disorder (MBD) develops as a systemic disorder of mineral and bone metabolism, including laboratory abnormalities such as parathyroid hormone (PTH), vitamin D, calcium, phosphate, fibroblast growth factor 23 (FGF-23), bone abnormalities, and vascular calcification [1]. Recent studies have illustrated that the elevated FGF 23 level induced by hyperphosphatemia and secondary hyperparathyroidism (SHPT) has direct adverse effects on cardiac myocytes and is associated with left ventricular hypertrophy [2,3]. CKD-MBD occurs early and increases in severity as CKD progresses to end stage renal disease (ESRD), resulting in high risks of bone fracture, cardiovascular diseases, and mortality [4].

In patients undergoing chronic hemodialysis (HD), SHPT is notably responsible for substantial morbidity and mortality related to renal osteodystrophy and vascular calcification [5]. The Kidney Disease Improving Global Outcomes (KDIGO) 2017 recommended that the patients with CKD stage 5D require PTH-lowering therapy including calcimimetics, calcitriol, and vitamin D analogs. However, dialysis patients with severe SHPT and extremely high PTH level are generally refractory to the medical PTH-lowering therapy stated above and usually require surgical parathyroidectomy (PTX) [6].

The indications for surgical PTX, as suggested by the different guidelines, are still inconclusive. The Japanese Society for Dialysis Therapy recommended surgical PTX for severe SHPT, PTH > 500 pg/mL, refractory to medical treatment [7]. The National Kidney Foundation-Dialysis Outcomes Quality Initiative (NKF-DOQI) suggested that surgical PTX should be recommended in patients with severe SHPT, persistent serum levels of PTH >800 pg/mL, associated with hypercalcemia and/or hyperphosphatemia that are refractory to medical therapy [8]. From Thailand renal replacement therapy registry, there were approximately 8–10% of dialysis patients with severe hyperparathyroidism, defined by serum intact parathyroid level more than 800 pg/mL. Finally, the KDIGO guidelines recommended surgical PTX in the patients with CKD stages 3–5 D with severe SHPT who fail to respond to medical/pharmacological therapy [6]. However, the KDIGO guidelines did not indicate the decisional levels of PTH for undergoing surgical PTX.

Cinacalcet is a calcimimetic agent that acts by activating calcium-sensing receptor in the parathyroid gland to lower the serum PTH level. Many randomized control trials demonstrated that cinacalcet can increase achievement of treatment target when compared with the conventional therapy [9,10]. Very few studies showed the effectiveness of cinacalcet in lowering PTH level and reducing parathyroid gland volume in severe SHPT with the PTH levels ranged >500–1200 pg/mL [11–13]. However, FGF-23, novel bone biomarkers, and vascular calcification score were not determined in these severe SHPT patients.

The present study was conducted in chronic HD patients with severe SHPT to examine the effectiveness and safety of cinacalcet, compared with the control group, on lowering PTH levels and to assess the regulatory roles of cinacalcet on CKD-MBD related parameters including FGF 23, novel bone biomarkers, size of parathyroid gland, and vascular calcification score.

## Methods

### Study design and population

This study was performed in two phases. Forty-five adult HD patients with severe SHPT were enrolled during October 2013–March 2014. In phase 1, a single center prospective randomized, open label study was conducted in 30 patients to compare the efficacy of cinacalcet (15 patients) versus the control group (15 patients) to explore the benefit on the achievement of >30% reduction of intact PTH (iPTH) from baseline as the primary end point regarding the efficacy assessment from the previous studies [14,15]. In phase 2, the 15 patients in the control group were switched to receive cinacalcet and 15 more new HD patients with severe SHPT were enrolled to achieve the total of 45 patients. All 45 participants were then followed as a prospective cohort study for 24-week follow up and 12 weeks after discontinuing cinacalcet to further explore the effect on CKD-MBD parameters including laboratory markers [calcium, phosphate, iPTH, FGF-23, bone alkaline phosphatase (BAP), vitamin D, 1,25 vitamin D, serum tartrate-resistant acid phosphatase 5 b (TRAP-5b), parathyroid gland size, and vascular calcification. The study was approved by the Institutional Review Board of the Faculty of Medicine, Chulalongkorn University, Bangkok, IRB 383/56 with clinical trial registration (NCT02056730). All participants received information of study details before giving written informed consent.

The inclusion criteria included age ≥18 years at screening, patients who were treated with maintenance HD at least 3 times a week for >3 months prior to screening, had plasma iPTH levels >800 pg/mL during screening, and had serum corrected total calcium >9.0 mg/dL. The patients who had low vitamin D level before enrollment had been prescribed nutritional vitamin D. Exclusion criteria were patients who had history of previous surgical PTX, had history of seizure within 12 weeks prior to randomization, had been scheduled for kidney transplantation, had been anticipated for surgical PTX within the next 6 months, had the results of liver function tests > than 2 × the upper limit of normal, had history of prior use of bisphosphonates, had been expected to receive bisphosphonates during the trial, and had active malignancy, pregnancy, breast feeding, and active infection.

### Study intervention

The permuted-block randomization was employed and the subjects randomized to treatment with cinacalcet (Regpara®; Ohara Pharmaceutical, Japan) received an initial oral dose of 25 mg once daily at bedtime. The dose was then adjusted within a range of 25–75 mg once daily based upon periodic measurements of serum calcium and iPTH levels. If no improvement was found in iPTH, the dose could be increased up to 100 mg once daily. If the dose increase was required, the dose should be increased by 25 mg at a time, at intervals of at least 3 weeks. If the serum Ca level fell below 8.4 mg/dL during the administration, the dose would not be increased and cinacalcet was withdrawn immediately when the Ca level is below 7.5 mg/dL. Regarding the concomitant drugs, the dose modification of vitamin D sterols included nutritional and active vitamin D was inhibited by rule during the study for both groups. Phosphate binder dose modification was allowed at the physician’s discretion during the study for both groups. Dialysate calcium concentration modification was allowed by rule during the entire study period. The radiologist who read the size of parathyroid gland by ultrasound measurement and vascular calcification that measured by x-ray lateral abdomen was blinded regarding the intervention of treatment.

### Biochemistry measurement

All serum biomarkers were measured by a central laboratory (Chulalongkorn Biochemical Research Laboratory, Bangkok, Thailand). The laboratory measured iPTH levels using a chemiluminescence immunoassay on a Roche Elecsys 2010 Analyzer; this assay detects both iPTH levels and a fragment containing amino acids 7–84; the reference range is 15.0–65.0 pg/mL. TRAP-5b levels were measured by Immunoassay (The MicroVue TRAP5b, Quidel Corporation, San Diego, USA). The normal range is 1.03–4.15 U/L in premenopausal women. The Intra-assay and inter-assay coefficients of variation (CVs) were 4.2% and 2.8%, respectively. Bone specific alkaline phosphatase (B-ALP) levels were measured by using an immunoassay (The MicroVue BAP, Quidel Corporation, San Diego, USA). The range of normal values was 15.0–41.3 U/L for males aged ≥25 years, 11.6–29.6 U/L for premenopausal females aged 25–44 years, and 14.2–42.7 U/L for postmenopausal females aged ≥45 years. The Intra-assay and inter-assay CVs were 5.5% and 5.0%; respectively. Fibroblast growth factor-23 (FGF-23) levels were measured using a Human intact FGF-23 ELISA kit (Millipore Corporation, Billerica, MA, USA). The Millpore lowest limit of detection was 3.5 pg/mL with an Intra-assay and inter-assay CVs of less than 11%. 25-OH vitamin D levels were measured by using chemiluminescent immunoassay (CLIA, Diasorin, Italy). Active vitamin D (1,25‐(OH)2D) levels were measured by using commercialized ELISA kits from Immundiagnostik, Bensheim, Germany. The Intra-assay and inter-assay CVs were 6.7% and 9.0%; respectively.

### Data collections

All patients were examined at seven points (baseline, week 3, week 6, week 9, week 12, week 24 and week 36 after cinacalcet initiation). For the drop-out cases: data of the end of study were collected. In phase 1, the primary endpoint was an achievement of >30% reduction of iPTH from the baseline while the secondary end points were the absolute values over time and the percentage changes in iPTH, P, Ca, BAP, TRAP-5b, FGF-23; and the absolute values over time and the percentage changes in size of parathyroid gland and vascular calcification in phase 2.

### Statistical analysis

The continuous variables were reported as mean ± standard deviation or median (interquartile). Categorical variables were reported in term of frequency and percentage. For normally distributed variables, the differences in changes between the two groups were analyzed by the unpaired *t*-test. The differences in changes within groups were analyzed by the paired *t*-test. For non-normal distributed variable, Mann-Whitney *U*-test was used to analyze the difference between the two groups. Categorical outcomes were analyzed by chi-square or Fisher exact test, as appropriate. The statistical analyses were performed by using the SPSS version 16.0 statistical software program.

## Results

**Figure 1.**
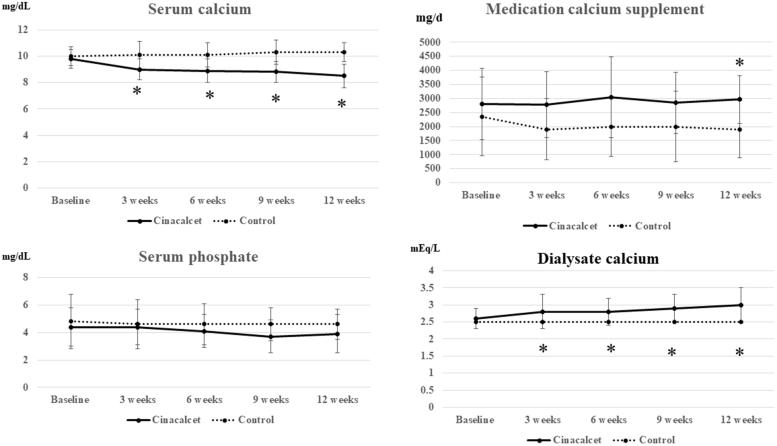
The change of serum calcium, serum phosphate, oral medication calcium supplement and dialysate calcium following baseline, 3 weeks, 6 weeks. 9 weeks, and 12 weeks in cinacalcet and control groups in Phase 1. *p < .05 when compared with control group.

**Figure 2.**
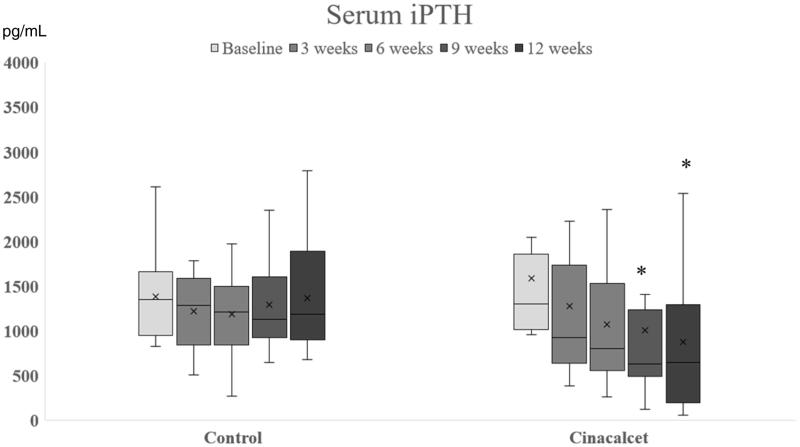
The change of serum iPTH following baseline, 3 weeks, 6 weeks, 9 weeks, and 12 weeks in cinacalcet and control groups in Phase 1. *p< .05 when compared with control group.

In phase 1, 30 participants were enrolled and randomly assigned to the cinacalcet (*n* = 15) or placebo (*n* = 15) group. The mean age and dialysis vintage were comparable between the two groups (49.4 ± 10.2 years, 7.1 ± 5.0 years in the control group vs 49.5 ± 11.9 years, 6.6 ± 3.6 years in the cinacalcet group). The baseline serum iPTH levels between the two groups were not different [1374 (955, 1639) pg/mL in the control group vs. 1191 (1005, 1884) pg/mL in the cinacalcet group)]. At 3-week and 6-week follow up, the mean serum calcium was significantly decreased in the cinacalcet group, the dialysate calcium was increased, and there was a lowering trend of serum iPTH levels (Figures 1 and 2). The significantly decreased serum iPTH levels were observed at 9-week and 12-week follow up (Figure 2). Following 12-week treatment, the percentage of patients achieving the target iPTH were significantly different between both groups [80% in the cinacalcet group (12/15 HD patients) and 13% in the control group (2/15 HD patients), *p* = .001].

**Table 1.** CKD-MBD Parameters during the second phase of study.

CKD-MBD parameters in phase 2 of study (*n* = 45)	At baseline	Week 24 of cinacalcet treatment	*p*-Value (baseline vs. week 24)	Week 36 (12 weeks after cinacalcet discontinuation)	*p*-Value (week 24 vs. week 36)
Serum iPTH (pg/mL)	1495(1024.50, 2039.50)	639.85(231.13, 1,510.75)	<.001	1352(655.55, 1861.75)	<.001
Serun FGF-23 (pg/mL)	1467.84(235.13, 8895.10)	158.70(34.57, 540.10)	<.001	703.14(113.34, 3428.63)	<.001
Serum TRAP-5b (ng/mL)	3.23(0.73,4.59)	2.87(1.25, 5.01)	.94	4.39 (1.87, 6.73)	.64
Serum BAP (ng/mL)	87.00(40.00, 309.00)	90.00(50.00, 362.00)	.05	91.50(39.75, 376.75)	.17
Serum calcium (mg/dL)	9.92 ± 0.84	9.00 ± 1.04	<.001	10.16 ± 1.07	<.001
Serum phosphate (mg/dL)	4.65 ± 1.66	3.65 ± 1.39	.001	4.96 ± 2.22	<.001
Dialysate calcium (mEq/L)	2.64 ± 0.35	3.26 ± 0.44	<.001	2.75 ± 0.44	<.001
Total calcium intake (mg/day)	923.12 ± 424.71	1,103.70 ± 715.48	.49	817.39 ± 643.61	.02
Serum 25(OH)D (ng/mL)	28.41 ± 11.93	26.93 ± 11.89	.24	21.56 ± 9.05	.001
Serum 1,25(OH)2D (ng/mL)	18.00 (14.00, 26.00)	13.00 (8.00, 21.00)	.01	10.50 (6.00, 18.25)	.08

BAP: bone alkaline phosphatase; iPTH: intact parathyroid hormone; FGF-23: fibroblast growth factor-23; TRAP-5b: tartrate-resistant acid phosphatase 5b; 25(OH)D: 25-hydroxy vitamin D; 1,25(OH)2D: 1,25-dihydroxy vitamin D.

In phase 2, 45 patients were followed up as a prospective cohort study for 24 weeks to further explore the effect of cinacalcet on CKD-MBD parameters including laboratory markers, such as calcium, phosphate, iPTH, FGF-23, BAP, vitamin D, 1,25 vitamin D, TRAP5b; parathyroid gland size; and vascular calcification at 24 week-follow up and 12 weeks after discontinuing cinacalcet.

In the phase 2 prospective cohort, the mean age of the 45 participants was 47.7 ± 12.4 years. The mean serum calcium, phosphate, and dialysate calcium levels were 9.92 ± 0.84 mg/dL, 4.65 ± 1.66 mg/dL, and 2.64 ± 0.35 mEq/L, respectively. The mean total calcium medication intake was 923.12 ± 424.71 mg/day. The mean dose of cinacalcet was 50.00 ± 23.23 mg/day. When the participants were divided into 3 groups based on their baseline iPTH levels, the percentage of those whose serum iPTH achieved the target of 130–585 pg/mL was significantly decreased 42%, 27%, and 0% in the groups with the baseline PTH levels of 800–1600, 1600–2400, and over 2400 pg/mL, respectively.

The CKD-MBD parameters observed during the phase 2 were presented in Table 1. The serum calcium, phosphate, iPTH, and dialysate calcium levels were significantly decreased from the baseline to 24-week follow up and were significantly increased after discontinuing cinacalcet. The similar trend was demonstrated on the median serum FGF-23. Almost 80% of the patients had decreased serum FGF-23 more than 30% from the baseline. Serum TRAP-5b had a trend to decrease from the baseline to 24-week follow up and was significantly increased after stopping cinacalcet. However, the median serum BAP did not have significant changes during the follow up periods. The mean serum 25(OH)D and 1,25 (OH)2 D had a trend to decrease along the timing of follow up (Table 2). Regarding the side effects, there were no serious adverse effects in both phases.

**Table 2.** Characteristics of participants in cinacalcet and control groups at baseline, and week 12 of follow-up in phase 1.

Phase 1 of study	Control (*N* = 15)	Cinacalcet (*N* = 15)	*p*-Value
Age (mean ± SD, years)	49.4 ± 10.2	49.5 ± 11.9	.99
Male (number (%))	7 (47%)	12 (80%)	.06
Hemodialysis duration (mean ± SD, years)	7.1 ± 5.0	6.6 ± 3.6	.75
Baseline			
• Serum calcium (mean ± SD, mg/dL)	10.0 ± 0.7	9.8 ± 0.7	.41
• Serum phosphate (mean ± SD, mg/dL)	4.8 ± 2.0	4.4 ± 1.4	.45
• Serum iPTH (pg/mL)	1374 (955, 1639)	1191 (1005, 1884)	.11*
• Serum 25(OH) (mean ± SD, ng/mL)	26.5 ± 13.2	22.9 ± 11.8	.44
• Serum FGF-23 (pg/mL)	1692 (652, 3958)	1434 (180, 4980)	.74
• Serum TRAP-5b (u/L)	1.2 (0.6, 2.0)	1.7 (0.4, 4.2)	.37
• Serum albumin (mean ± SD, g/dL)	4.3 ± 0.4	4.5 ± 0.5	.17
• Dialysate calcium (mean ± SD, mEq/L)	2.5 ± 0.0	2.6 ± 0.3	.19
• Oral medication calcium treatment (mean ± SD, mg/d)	943 ± 562	1117 ± 508	.62
• Active vitamin D supplement (mean ± SD, ug/week)	3.5 ± 3.1	3.3 ± 0.9	.67
Week 12			
• Serum calcium ( mean ± SD, mg/dL)	10.3 ± 0.7	8.5 ± 0.9	<.001
• Serum phosphate ( mean ± SD, mg/dL)	4.6 ± 1.1	3.9 ± 1.4	.12
• Serum iPTH (pg/mL)	1199 (918, 1877)	565 (184, 1067)	.022*
• Serum 25(OH) (mean ± SD, ng/mL)	26.0 ± 4.9	23.0 ± 8.6	.25
• Serum FGF-23 (pg/mL)	1252 (294, 2371)	243 (34, 555)	.043*
• Serum TRAP-5b (u/L)	1.9 (1.0, 3.6)	1.7 (0.1, 2.9)	.70
• Serum albumin ( mean ± SD, g/dL)	4.3 ± 0.4	4.2 ± 0.5	.83
• Dialysate calcium ( mean ± SD, mEq/L)	2.5 ± 0.0	3.0 ± 0.5	.001
• Oral medication calcium treatment ( mean ± SD, mg/d)	760 ± 410	1185 ± 341	.04
• Active vitamin D supplement (mean ± SD, ug/week)	3.5 ± 2.9	3.3 ± 1.0	.85

25(OH): 25-hydroxy vitamin D; FGF-23: fibroblast growth factor 23; iPTH: intact parathyroid hormone; TRAP-5b: tartrate-resistant acid phosphatase 5b *nonparametric Mann-Whitney *U*-test.

The slightly decreased size of parathyroid gland from 1.21 (0.25, 3.69) cm^3^ to 1.01 (0.18, 3.71) cm^3^ was also demonstrated. Fifty-five percent of patients had reduced size of parathyroid gland and this was significantly associated with more than 30% decrease in serum iPTH level from the baseline (*p* = .013). The median vascular calcification score was 6.0 (0.0, 13.0) at baseline and 5.0 (0.3, 12.8) after 24-week follow up. The decreased vascular calcification was observed around 20%, while 65% of the participants had stable vascular calcification, and only 15% had progression of vascular calcification during the 24-week follow up.

## Discussion

In this current study, the efficacy of cinacalcet on severe SHPT in chronic HD patients was explored. Serum iPTH, calcium, phosphate, 25-OH vitamin D, 1,25-(OH)2 vitamin D, and FGF-23 together with bone markers such as TRAP-5b and BAP were investigated among HD patients with the median serum iPTH of around 1400 pg/mL. In phase 1, the significant reduction of iPTH was observed after the third visit (9-week follow-up) (Figure 2). Although the dialysate calcium concentration was adjusted to reduce the risk of hypocalcemia, the significant decreasing of serum calcium from the baseline was observed since the first follow-up visit (week 3) (Figure 1). In the second phase of study, the CKD-MBD markers at week 24, namely: iPTH, and FGF-23, were significantly decreased from the baseline and markedly rebound at week 12 after discontinuation of cinacalcet (Table 1), suggesting that the long term treatment is needed for controlling these disorders. The achievement target of iPTH around 40% was still observed in the HD patients with the baseline serum iPTH levels around 800–1600 pg/mL but the percentage of patients who achieved the iPTH target was lower among those with higher serum iPTH at the baseline, implying that those who had serum iPTH levels of higher than 1600 pg/mL should be advised to undergo surgical PTX.

The SHPT is a severe complication of patients on chronic HD and is responsible for substantial morbidity and mortality related to osteodystrophy and vascular calcification [16–18]. The hyperplasia of parathyroid glands is initially polyclonal diffused and successively reversible controlled. The nodular or adenomatous parathyroid glands begins to function autonomously and continue to secrete PTH even if hypocalcemia is corrected. This condition is referred to as ‘refractory’ SHPT and is also sometimes called ‘tertiary’ hyperparathyroidism (THPT) [19]. Prior to the cinacalcet era, it was shown that the calcium nonsuppressible PTH secretion correlates with the degree of parathyroid gland enlargement and the severity of SHPT [20]. Indeed, the parathyroid gland hyperplasia is associated with a progressive down-regulation of calcium sensing and vitamin D receptors [21–24]. The receptor downregulation may influence the proliferation of hyperplasia and it may explain the unresponsiveness to drug therapy [25,26]. Thus, when nodular hyperplasia develops, drug therapy is more likely to fail and at this point of transformation from SHPT to THPT, PTX may be indicated [27]. This study demonstrated the effectively decreasing of serum iPTH and had a trend of decreased parathyroid gland size in patients with the baseline serum iPTH levels around 800–1600 pg/mL. These findings suggest that chronic HD patients who had serum iPTH levels higher than 1600 pg/mL should be advised to surgical PTX. In addition, although substantial and expected declines in laboratory values occurred following cinacalcet initiation, but rebounding of all laboratory makers were observed in this study (Table 1). In agreement with the present study, a previous study also observed that early discontinuation due to economic reasons caused increases in laboratory markers again [28].

Before the cinacalcet era, the volume or size of parathyroid gland, indicating nodular hyperplasia, was undoubtedly an important factor in predicting the future responsiveness to vitamin D or analog treatment. Instead, in regard to therapy with cinacalcet, Komaba et al. reported that cinacalcet could effectively decrease serum iPTH levels and concomitantly reduced parathyroid gland volume in the patients who had serum iPTH around 500–800 pg/mL during 1-year follow up [29]. Yamada et al. reported that cinacalcet treatment with intravenous VDRA therapy decreased both parathyroid gland volume and serum iPTH level during 2-year follow up in the patients who had baseline iPTH 300–800 pg/mL [30]. Meola et al. reported on nine patients who had serum iPTH around 1200 pg/mL and 24–30 months of follow up, cinacalcet could reduce glandular volume [31]. The slightly decreased size of parathyroid gland was also observed in the present study. The severity of disease and short term follow up might explain the observed results.

Serum FGF-23 is associated with left ventricular hypertrophy and has been linked to an increased risk for cardiovascular mortality [2,3,32]. Although a recent meta-analysis demonstrated that cinacalcet can decrease serum FGF-23, most included studies were performed in nonsevere SHPT (serum iPTH levels below 1000 pg/mL) [13]. The present study illustrated that cinacalcet can still reduce serum FGF-23 in chronic HD patients with the median serum iPTH levels of around 1400 pg/mL (Table 1). However, the long term benefit of this finding on the left ventricular mass index, cardiovascular morbidity, and mortality needs further studies.

Cinacalcet could stabilize vascular calcification in our study, and only fifteen percent of patients had progression of vascular calcification. Of note, there was only one previous trial of 190 patients that reported on the effect of cinacalcet combined with vitamin D on vascular calcification, and demonstrated a significant decrease in the calcification of coronary arteries and the aortic valve on both the Agatston and volume score relative to the control group (vitamin D alone) during 1-year follow up [33].

Although surgical PTX can effectively decrease the iPTH levels in the majority of patients, the improvement in the biochemical control following surgical PTX may not be perfect. Indeed, there is a relatively high failure rate for an elective surgical PTX, possibly related to surgical and center experience. Regardless of the surgical technique employed, the recurrence rates were around 10% in the long term [34]. With much interest in FGF-23, it is notable that no consistent data show the changes in these values post-PTX [35,36]. Finally, a recent meta-analysis demonstrated that surgical PTX significantly decreased cardiovascular mortality and all-cause mortality in observational studies [37]. However, the interpretation of the results should be careful due to the selection bias, and there was no controlled arm such as medical therapy.

Our study has some limitations. First, the number of our studied population was quite small. Although the significance of cinacalcet effects on iPTH and FGF-23 was obviously illustrated, the present study probably did not have sufficiently statistical power to detect any differences (if one existed) in the changes of TRAP-b5 and BAP among studied time-points. Second, in term of the bone markers, cinacalcet decreased the bone resorption markers, suggesting that cinacalcet might also decrease the bone turnover. However, the effects of cinacalcet on the bone density and the incidence of fracture were not examined in the present study. The impact of cinacalcet on these clinical outcomes requires further investigations.

In conclusion, the effectiveness and safety of cinacalcet were still apparently demonstrated even in case of severe SHPT. Some patients might be able to avoid or postpone the surgical PTX. The improvement of bone markers and FGF-23, and the stabilization of vascular calcification were also observed. Therefore, cinacalcet can provide salutary effects on CKD-MBD in chronic HD patients with severe SHPT and might be an initially effective PTH-lowering therapy prior to surgical PTX as well as an alternative treatment in the patients unsuitable for surgical PTX.

## Disclosure statement

No potential conflict of interest was reported by the authors.
